# Neuronal nitric oxyde synthase positive neurons in human indusium griseum

**DOI:** 10.1007/s00429-022-02484-z

**Published:** 2022-04-08

**Authors:** Teresa Lorenzi, Andrea Sagrati, Eva Montanari, Martina Senzacqua, Manrico Morroni, Mara Fabri

**Affiliations:** 1 Department of Experimental and Clinical Medicine, Section of Neuroscience and Cell Biology, School of Medicine, Università Politecnica delle Marche, Via Tronto 10/A, 60126 Ancona, Italy; 2 Department of Biomedical Sciences and Public Health-Legal Medicine Unit, School of Medicine, Università Politecnica delle Marche, Via Tronto 10/A, 60126 Ancona, Italy; 3 Electron Microscopy Unit, United Hospitals, Via Conca 71, 60020 Ancona, Italy; 4 Present Address: Department of Life and Environmental Sciences, Università Politecnica delle Marche, Via Brecce Bianche, 60131 Ancona, Italy

**Keywords:** Immunohistochemistry, Immunofluorescence, Western Blot, Nitric oxide, Neurovascular unit, Pial artery

## Abstract

The study was designed to analyze the nNOS positive neurons present in the indusium griseum by describing their distribution and morphology. To this purpose, sagittal serial sections from paraffin or frozen autopsy specimens of corpus callosum including the overlying indusium griseum were processed by immunohistochemistry and immunofluorescence, using an antibody against the neuronal form of the enzyme nitric oxyde synthase. To test the specificity of the antibody used, Western Blot was performed in the indusium griseum of the same specimens. The stainings revealed the presence of many neuronal nitric oxyde synthase-immunopositive neurons in human indusium griseum, located along both rostral-caudal and medio-lateral directions. In particular, they were more numerous 1 mm apart from the midline, and their number peaked over the body of the corpus callosum. They showed different morphologies; in some cases, they were located at the boundary between indusium griseum and corpus callosum, more densely packed in proximity to the pial arteries penetrating into the corpus callosum. The significant presence and distribution of neuronal nitric oxyde synthase-immunopositive neurons suggests that indusium griseum likely plays a functional role in the neurovascular regulation within the corpus callosum.

**Graphical abstract:**

Schematic representation of human adult IG and the neurovascular unit originating from sopracallosal artery (Sca) that branches into smaller arterioles (Br) (created in PowerPoint). The arterioles cross the three layers of IG (layers I, II and III) and penetrate into the CC separated from IG by the Virchow-Robin space (VRs). As the arterioles go deeper, this space disappears and the vascular basement membrane comes into direct contact with the astrocytic end-feets (intracallosal arterioles and capillaries). nNOS-immunopositive neurons (nNOS_IP_ N) surround the arterioles and control the vasomotore tone secreting nitric oxyde (NO). Two morphological types of nNOS_IP_ N can be appreciated by the use of different colors: fusiform (blue) and ovoidal (pink). Also NeuN-immunopositive neurons (N) and many astrocytes (As) are present, more numerous in IG than in CC.

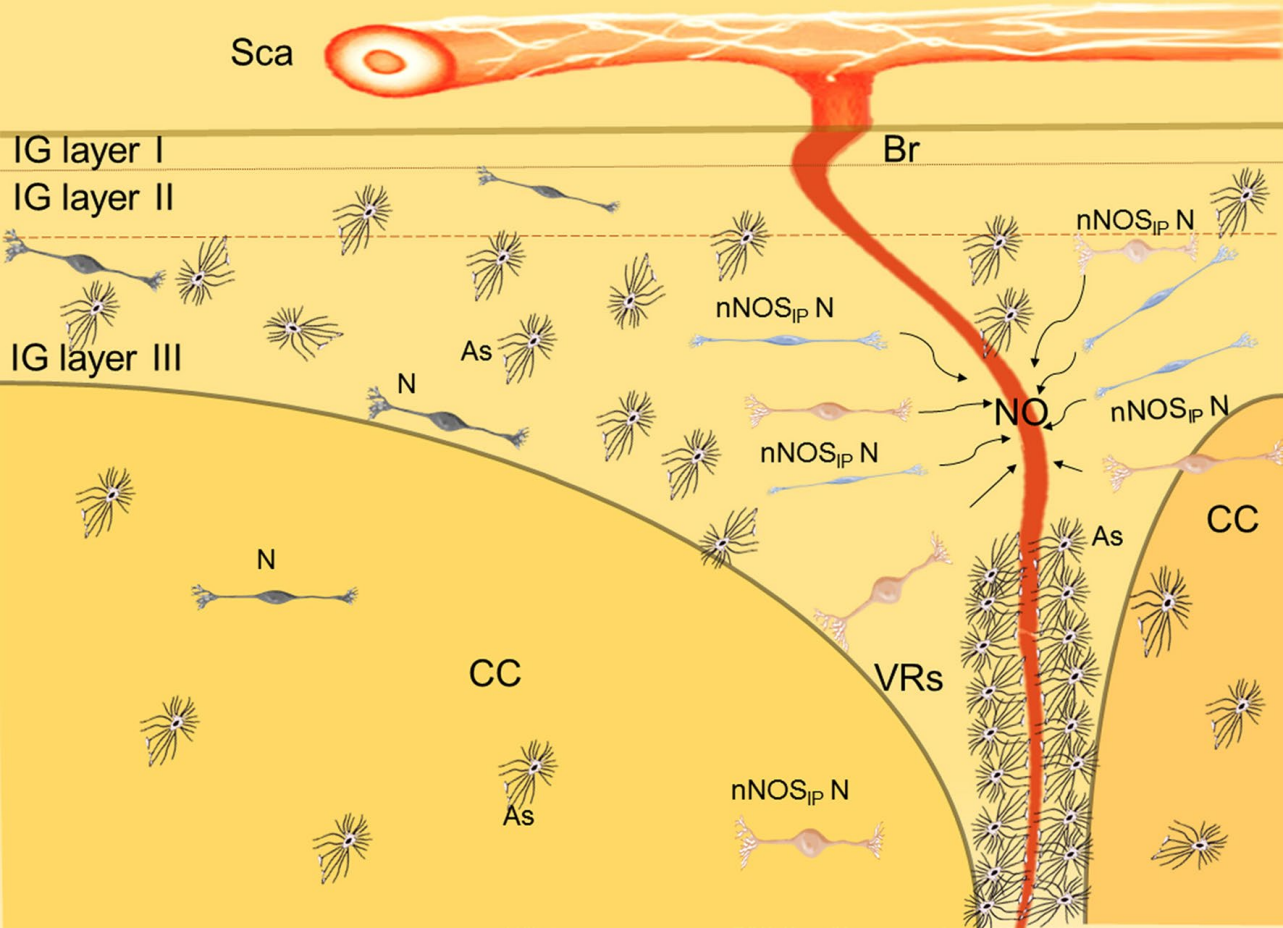

## Introduction

The Indusium Griseum (IG) is a thin neuronal lamina above the corpus callosum (CC), consisting of two layers of gray matter (Laplante et al. 2013; Di Ieva et al. 2015). The IG continues rostrally toward the ventral portion of the genu of the CC and dorsally to the taenia tecta. It has been considered a component of the limbic system (Di Ieva et al. 2015), but whether IG is an embryological remnant or an active functional element it is still unclear. A very recent paper in humans (Bobić Rasonja et al. 2021) found that IG is developmentally distinct from the hippocampal formation; in addition, it exhibits continuous maturation, also in the final trimester of gestation, without signs of regression during the fetal period; this suggests that IG is not a rudimentary tissue, but may play a functional role in the adult brain (Bobić Rasonja et al. 2021). Moreover, a Golgi (Wyss and Sripanidkulchai 1983) and an immunocytochemical study (Barbaresi 2018) described the presence of neurons within the rat IG. In accordance with previous studies, Lorenzi et al. (2019) confirmed that human IG contains neurons, and that some of them are immunopositive to neuronal nitric oxide synthase (nNOS), the enzyme responsible for the synthesis of nitric oxide (NO). Nitric oxide is a neurotransmitter that participates in numerous physiological functions; it is synthesized by NO synthase (NOS) from l-arginine and, being a gaseous molecule, it is released by simple diffusion from nerve terminals. Nitric oxide is produced at least by three isoforms of NOS: neuronal (nNOS), inducible (iNOS), and endothelial (eNOS) (Dawson and Snyder 1994; Zhang and Snyder 1995). The physiological sphere of influence of NO has been estimated to have a radius of about 200–400 µm, corresponding to a brain volume enclosing 2 million synapses (Wood and Garthwaite 1994; Estrada and DeFelipe 1998; Laranjinha et al. 2012). Nitric oxide is one of the numerous neurotransmitters playing a key role in the regulation of cerebral blood flow (CBF), by modulating vascular tone. Cerebral BF is critically important for brain function and viability, delivering nutrients and oxygen (Fantini et al. 2016). When the activity of a brain region increases, BF to that region also increases. This mechanism, termed functional hyperaemia, controls not only the substrate and oxygen delivery, but also the removal of metabolism by-products (Iadecola 2004). The primary blood supply to the CC is provided by the callosal arteries which originate from the pericallosal artery (Kakou et al. 2000; Ugur et al. 2006). The callosal arteries give rise to perforating branches to the IG. Thus, nNOS-containing neurons of IG could be potentially involved in coupling local increases in perforating branches BF with metabolic changes related to neuronal function of the underlying CC (Jovanov-Milosevic et al. 2010; Sagrati et al. 2018, 2019). Given the potential crucial role of the IG in the control of callosal BF, we decided to study the IG nNOS-immunopositive neurons quantifing them and analyzing their distribution and morphology, with the aim to shed new light on IG as a very active anatomical structure.

## Materials and methods

### Tissue sources and preparation

Adult human autopsy brains were obtained from 20 subjects without known neurological pathologies and sequentially recruited. The gender, age at death, and postmortem-interval (PMI) (the time between death and removal of the brain tissue from the cranial cavity; PMI include the refrigeration of the corpses after their discovery) were registered, and reported in Table 1. To remove CC and overlying IG specimens, the interhemispheric fissure was exposed and gently retracted. The tissue samples were collected from both hemispheres, when available, and prepared for morphological and/or biochemical analysis. Samples with visible signs of decomposition were excluded. Samples for Western Blot (WB) analyses were immediately frozen in liquid nitrogen and stored at − 80 °C; those for morphological analyses were fixed for 11 days in 4% neutral buffered formalin at 4 °C. The latter samples were then either embedded in paraffin or frozen (Table 2).

**Table 1:** Data from selected cases

Case* n*	Gender	Age	PMI (h)
1	F	28	24
2	F	38	48
3	M	51	24
4	M	61	72
5	M	79	48
6	F	41	48
7	F	43	48
8	F	40	96
9	M	54	48
10	M	57	48
11	F	39	72
12	F	40	24
13	M	48	24
14	M	30	48
15	M	14	72
16	M	6	120
17	M	81	168
18	F	69	168
19	M	84	24
20	M	74	96

**Table 2:** Sampling details for the different morphological  analyses

Method	Kind of section	Section thickness	Cases studied	Blocks per case	Sections per block
Immunohistochemistry (qualitative)	Paraffin	15 μm	20	3 (anterior IG)	15
Immunohistochemistry (quantitative)	Frozen	60 μm	3	2 for case 6 (middle and posterior IG) 1 for case 7 (anterior IG) 3 for case 8 (anterior, middle and posterior IG)	125
Immunofluorescence (qualitative)	Paraffin	5 μm	20	1 (anterior IG)	10

All procedures for samples collections were approved by the Ethics Committee of Università Politecnica delle Marche, in accordance with the Helsinki Declaration of 1975, as revised in 2013. Samples were kept anonimous and exclusively linked to codes.

### Western Blot

Indusium griseum specimens for WB analysis were obtained from 5 cases in whom both hemispheres were available; they were separated from CC by mechanical detachment, immediately after the sample collection. Tissue lysates of IG were prepared by complete potter homogenization in ice (Ultra-Turrax T8, IKA-WERKE, Lille, France) in lysis buffer containing 1% Triton X-100, 50 mM HEPES, pH 7.5, 150 mM NaCl, 10% glycerol, 1.5 mM MgCl_2_, 5 mM EGTA and protease inhibitors (Pefabloc SC; Complete Roche Diagnostic S.p.A.), freshly added. As a control of the method used, rat CC tissue lysate known to contain nNOS protein (Barbaresi et al. 2014) was analyzed in WB. Protein concentrations were assessed with Bradford protein assay (Bio-Rad Laboratories, Milano, Italy). Equal amounts of proteins (500 µg) were denatured with 1× sample buffer, boiled for 5 min, and fractionated on 8% SDS-polyacrilamide gels (SDS-PAGE). Blots were first incubated with 5% BSA (Bovine Serum Albumin; Sigma, Milano, Italy) in TBS-T 0.5% and then overnight at 4 °C with policlonal anti-human nNOS antibody made in rabbit (Cayman Chemical, Hamburg, Germany) diluted 1:700 in TBS (Table 3). After washing, blots were incubated with anti-rabbit secondary antibody conjugated to horseradish peroxidase (Amersham Italia srl, Milano, Italy) (Table 4) diluted 1:5000 in TBS. Detection of bound antibody was performed with ECL-Western blotting detection kit (Amersham) according to the manufacturer’s instructions.

**Table 3:** Primary antibodies used in the study

Label	Antibody	Supplier/location	Polyclonal/monoclonal	Immunogen	Dilution (μl)	Catalog #	RRID
nNOS	Rabbit anti-human Neuronal Nitric Oxide Synthase	Cayman Chemical, Hambrug, Germany	Polyclonal	Synthetic peptide from the C-terminal region of human nNOS	1/700 for WB^a^ 1/500 for IHC^b^ and IF^c^	160870	AB_10080041
NeuN	Mouse anti-human Neuronal Nuclei	Merck, S.p.a., Milano, Italy	Monoclonal	Purified cell nuclei from mouse brain	1/100 for IHC and IF	MAB 377	AB_177621
α-SMA	Mouse anti-human alpha-Smooth Muscle Actin	Agilent Dako, Santa Clara, CA, USA	Monoclonal	SDS extracted protein fraction of human myocardium	1/100 for IHC and IF	M0635	AB_2242301
GFAP	Goat anti-human Glial Fibrillary Acidic Protein	Merck, S.p.a., Milano, Italy	Polyclonal	Peptide with sequence C-DGEVIKESKQEHKD from the C-terminal of the protein sequence according to NP_002046.1	1/100 for IF	SAB2500462	AB_10603437

^a^Western Blot

^b^Immunohistochemistry

^c^Immunofluorescence

**Table 4:** Secondary antibodies used in the study

Label	Antibody	Supplier/location	Dilution (μl)	Catalog #	RRID
Horserdish peroxidase	Donkey anti-Rabbit	Amersham Italia srl, Milano, Italy	1/5000	NA934	AB_772206
Biotinylated	Goat anti-Rabbit	Vector Laboratories, Burlingame, CA	1/200	BA-1000	AB_2313606
Biotinylated	Goat anti-Mouse	Vector Laboratories, Burlingame, CA	1/200	BA-9200	AB_2336171
Alexa Fluor^®^ 488	Goat anti-Rabbit	Abcam, Cambridge, UK	1/200	ab150077	AB_2630356
Alexa Fluor^®^ 555	Goat anti-mouse	Abcam, Cambridge, UK	1/200	ab150114	AB_2687594
Alexa Fluor^®^ 647	Donkey anti-goat	Abcam, Cambridge, UK	1/200	ab150131	AB_2732857

### Immunohistochemistry

Qualitative analysis was performed in thin paraffin sections; frozen thicker sections were used for quantitative analysis.

#### Paraffin sections

Small tissue blocks of IG overlying callosal genu from all 20 cases were embedded in paraffin and cut in 15-µm-thick adjacent sections in sagittal plane using a sliding microtome (Reichert, Austria). Paraffin sections were deparaffinized and rehydrated via xylene and a graded series of ethyl alcohol.

To delineate the boundaries and cellular compartments of the midline structures, sections were stained by Luxol fast blu (Dia-Path, Bergamo, Italy) and eosin (Dia-Path), allowing to identify the IG, as previously described (Lorenzi et al. 2019).

To inhibit endogenous peroxidase activity, sections were incubated for 30 min with 3% hydrogen peroxide in methanol and then washed in phosphate-buffered saline (PBS). For the detection of Neuronal Nuclear marker protein (NeuN), retreatment of the sections occurred in a water bath for 30 min at 90 °C in 0.1 M sodium citrate (pH 7.2), according to antibody datasheet. To block nonspecific background, the sections were incubated with BSA (Sigma) for 1 h at room temperature (RT).

Sections were then incubated overnight at 4 °C with one of the following primary antibodies: (a) anti-human nNOS policlonal antibody made in rabbit; (b) anti-human NeuN monoclonal antibody made in mouse; (c) anti-human alpha-Smooth Muscle Actin (α-SMA) (a marker for mature myofibroblasts) monoclonal antibody made in mouse. For antibodies dilutions and commercial suppliers, see Table 3.

After washing in PBS, the sections were incubated for 1 h at RT with biotinylated goat anti-rabbit (bGAR) or goat anti-mouse secondary antibody (bGAM) (Table 4). The Avidin–Biotin-Complex with peroxidase method (ABC; Vector Laboratories) was performed for 1 h at RT, and 3-3′ diaminobenzidine hydrochloride (DAB; Sigma-Aldrich, St Louis, MO, USA) was used as chromogen. Normal human renal macula densa tissue was used as positive control for nNOS (Lorenzi et al. 2020). Immunostained sections were inspected with a motorized Leica DM6000 microscope (Leica Microsystems, Wetzlar, Germany), using different magnifications (10×, 25× and 40×).

#### Frozen sections

Fixed tissues from three cases (6, 7 and 8) were cryoprotected in 20% sucrose in PBS, frozen at – 80 °C, and cut in 60-µm-thick serial sections on a sliding microtome (Reichert, n. 391102, Austria) from the midline to 5 mm laterally. Sections for immunohistochemistry were first transferred to a solution of 3% H_2_O_2_ in PBS for 30 min, to inhibit endogenous peroxidase activity, then incubated for 1 h in a blocking solution consisting of 2% BSA (Sigma) in PBS. After several rinsing in PBS, sections were incubated overnight at 4 °C in primary anti-human nNOS policlonal antibody made in rabbit (Cayman Chemical) (Table 3). After washing in PBS, sections were placed for 1 h at RT in bGAR secondary antibody (Vector Laboratories) (Table 4), then rinsed and reacted with freshly prepared ABC complex (Vector Laboratories). After several washes, sections were processed for peroxidase histochemistry using 0.02% DAB (Sigma-Aldrich) in 0.05 mM Tris buffer, pH 7.6. After a final rinse in PBS, sections were mounted on subbed slides, air-dryed, dehydrated, and coverslipped. A motorized Leica DM6000 microscope (Leica Microsystems) was used to analyze immunostained sections at different magnifications (10×, 25× and 40×). The brightness and contrast of the final images were adjusted using Photoshop CS6 (Adobe Systems, Mountain View, CA, USA) (RRID:SCR_014199).

### Immunofluorescence

This kind of analysis was performed on 5-µm-thick paraffin sections from 20 cases, immediately transferred onto coated slides (Super Frost Plus, Menzel-Glaser, Braunschweig, Germany). Sections were deparaffinized and rehydrated via xylene and a graded series of ethyl alcohol. One section from each paraffin block was stained with Rhodamine phalloidin (Merk S.p.a.), a high-affinity F-actin probe conjugated to the red–orange fluorescent dye tetramethylrhodamine (TRITC). Pretreatment occurred in a water bath for 30 min at 90 °C in 0.1 M sodium citrate (pH 7.2), according to NeuN datasheet. The slides were then incubated for 20 min at RT in 1% BSA (Sigma) in PBS and then at 4 °C overnight with 3 mixtures of the following primary antibodies (Table 3):

1. anti-human nNOS and anti-human NeuN;
2. anti-human αSMA and anti-human Glial Fibrillary Acidic Protein (GFAP; a marker for astrocytes);
3. anti-human nNOS, anti-human αSMA, and anti-human GFAP.

After overnight incubation, slides were washed in PBS and incubated for 1 h in the dark at RT, with a mixture of the following secondary antibodies (Table 4): (a) goat anti-rabbit IgG (Alexa fluor 488), (b) goat anti-mouse IgG (Alexa fluor 555) and (c) donkey anti-goat IgG (Alexa fluor 647). All sections were washed again in PBS, stained with Sudan Black to block the autofluorescence of the tissue and mounted in Vectashield Mounting Medium (Vector Laboratories). Fluorescence was detected with a Leica TCS-SL confocal microscope (Leica Microsystems) equipped with an Argon and He/Ne mixed gas laser. Images (pixels) were obtained sequentially from two channels using a confocal pinhole of 1.1200 and stored as TIFF files. The brightness and contrast of the final images were adjusted using Photoshop 6 (Adobe Systems).

### Quantitative analysis

Quantitative analysis of nNOS neurons was performed in immunohistochemistry processed frozen sections in the IG above callosal genu (anterior IG, cases 7 and 8), callosal body (middle IG, cases 6 and 8) and splenium (posterior IG, cases 6 and 8) (Table 5). The number of neurons expressing nNOS was counted per each section starting from the midline and proceeding laterally; sections were examined by a double blind procedure by two independent observers (T.L. and A.S.) and the nNOS positive neurons distribution was reproduced by means of a camera lucida attached to a Leitz Orthoplan microscope (Leitz, Wetzlar, Germany). We did not use the stereological counting because the presence of nNOS neurons in IG is not homogeneous and thus the estimation of their total number by optical fractionator was not possible.

A statistical analysis was not performed because of the small number of cases available for quantitative evaluation.

## Results

### Western Blot analysis

**Fig. 1:**
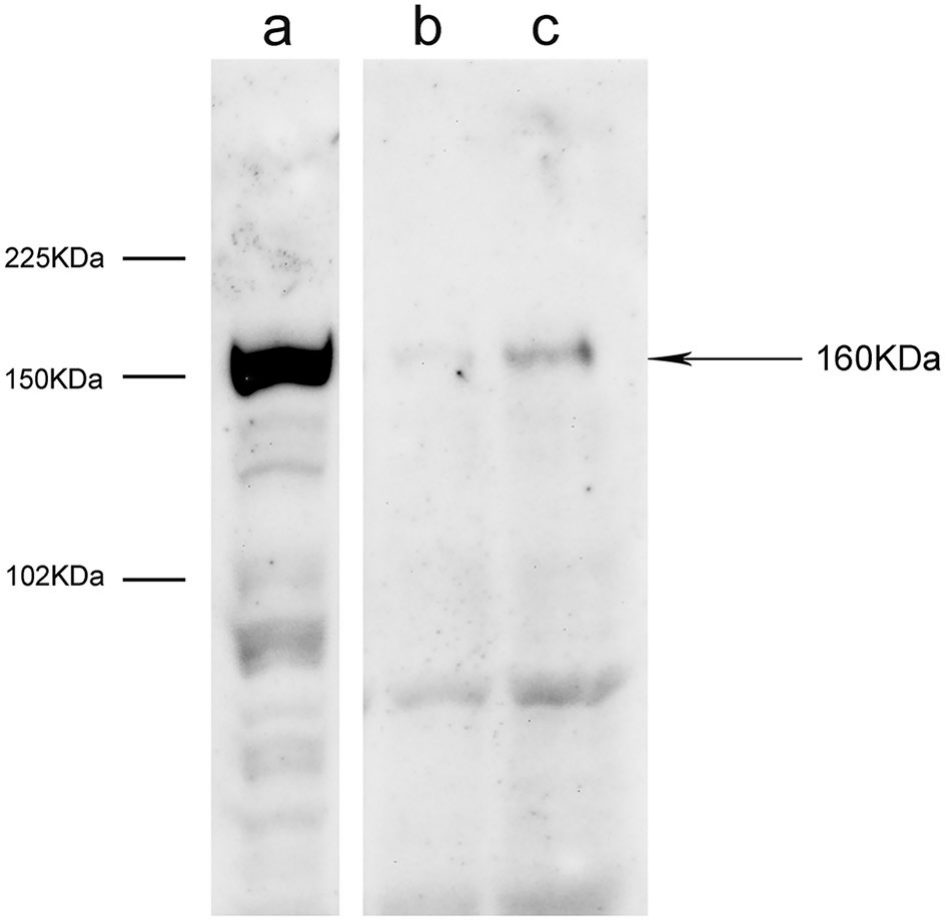
Cropped Western Blot image showing the presence of nNOS in the human IG. **a** Rat CC as control; **b** and **c** human IG tissue lysates from two cases (10, 11) on 8% SDS-polyacrilamide gel. The detected band shows the molecular weight of 160 kDa expected for nNOS.The blot retains at least six band width below the nNOS band. Lanes that were not contiguous in the experiment have a clear separation (vertical white space)

The nNOS antibody specificity was assessed in WB using rat CC tissue lysate sample as control: the staining revealed a unique positive band at the molecular weight (160 kDa) (Fig. 1a) typical of nNOS protein. The WB of human IG lysates was performed only from 2 fresh autopsy specimens (cases 10 and 11); after completing the tissue lysate preparation, a very small amount of protein was obtained from the 3 others samples, therefore they could not be analyzed; cases 10 and 11 revealed the presence of one band, more evident in case 11 (Fig. 1c) at the molecular weight typical of nNOS (160 kDa) (Fig. 1b, c).

**Table 5:** Absolute numbers and percentages of nNOS-positive neurons in the different IG zones above the CC

Cases	Number of nNOS-positive neurons			
	Anterior IG	Middle IG	Posterior IG	Total
6	NA^a^	98	55	153
7	86	NA	NA	86
8	188	725	173	1086
6 + 7 + 8	274	823	228	1325
Mean 6–8	137	412	114	663^b^
%	21	62	17	100

^a^Not available

^b^Value calculated by adding the means of anterior, middle and posterior IG

### Identification of nNOS-positive neurons by light and fluorescence microscopy

**Fig. 2:**
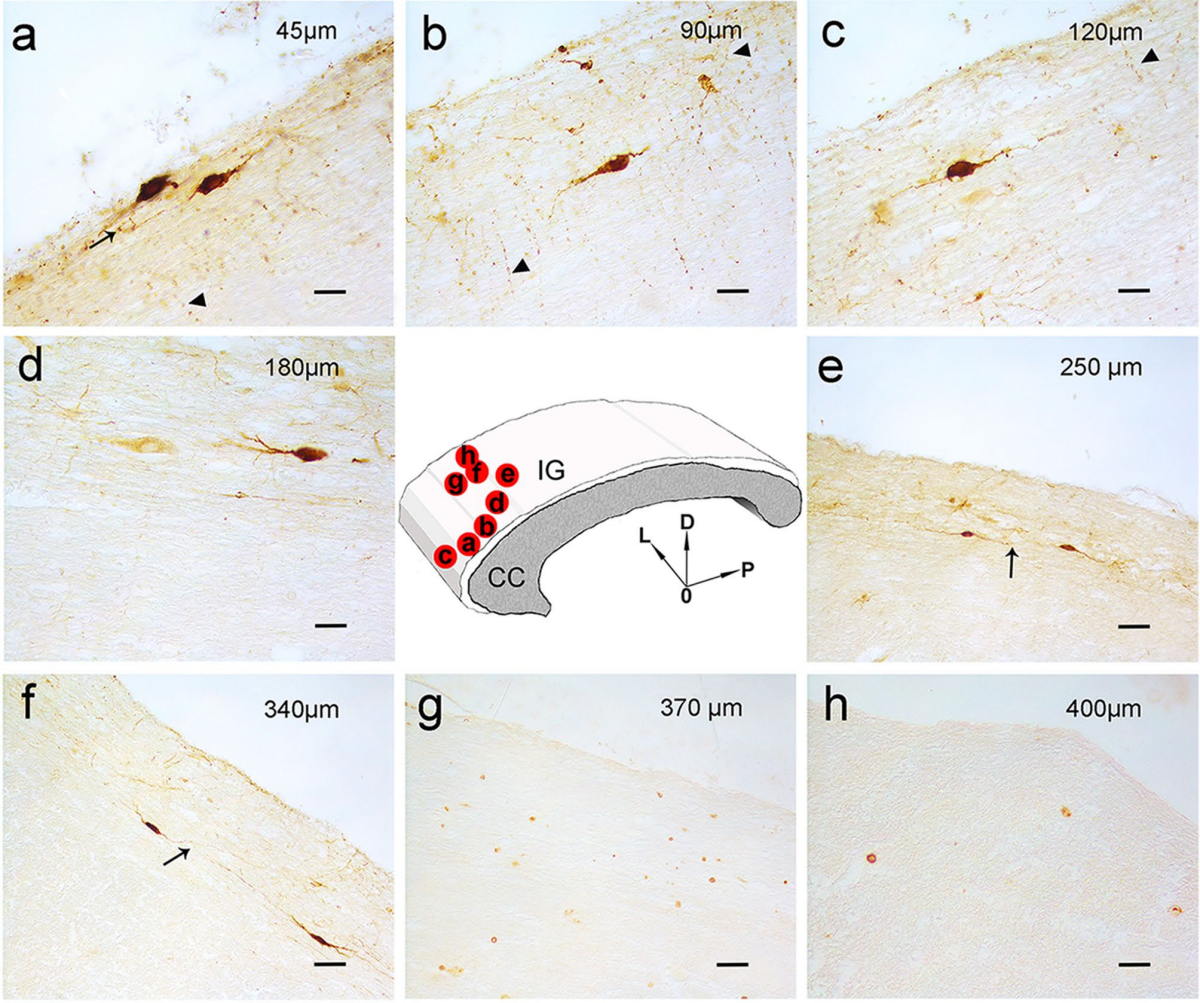
Photomicrographs of nNOS-immunopositive neuronal-like cells found in the anterior portion of the human IG. A schematic representation of CC and overlying IG is shown in the centre. The image in each panel was obtained from sagittal section whose location is indicated by a red circle in the central schema. In the upper right corner of each panel the distance of the section from the midline is indicated. **a**–**f** Photomicrographs showing different morphological types of nNOS-immunopositive cells: ovoidal (**a**–**d**), and fusiform (**e**, **f**). All cells are bipolar and horizontally oriented. Neuronal NOS positive fibers are indicated by arrow heads in **a**–**c**. Neuronal-like cells are close to each other (**a**, **e**, **f**, black arrows). **g**, **h**: NeuN-immunopositive nuclei in IG. *L* lateral, *D* dorsal, *P* posterior direction, *0* midline. Calibration bars: **a**–**c**, **d**, **h**, 25 µm; **e**–**g**, 50 µm

**Fig. 3:**
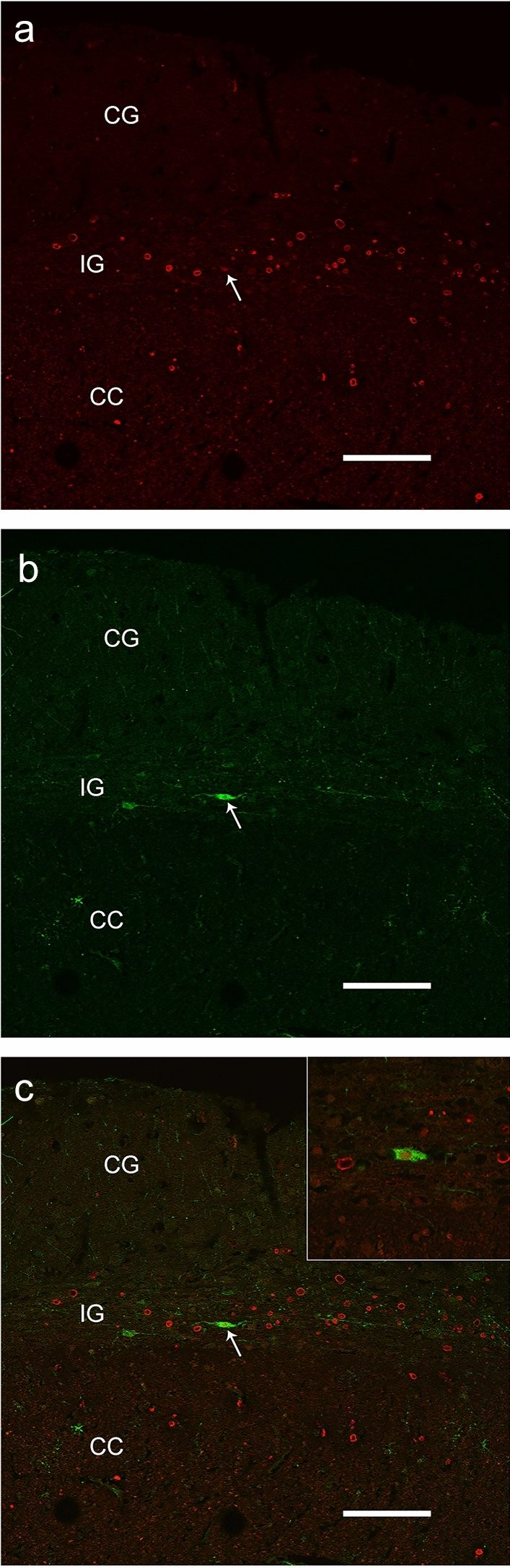
Confocal laser scanning photomicrographs. A NeuN-positive cell (**a** white arrow) is also positive to nNOS (**b** white arrow). **c** Merged image. The inset in frame c shows an nNOS positive neuron at higher magnification, in a slightly different plane of focus. Three layers, Cingulate Gyrus (CG), Indusium Griseum (IG) and Corpus Callosum (CC) can be distinguished. Calibration bars: 100 µm in **a**–**c**; 50 µm in the inset

Immunohistochemistry, performed using the anti-nNOS and anti-NeuN antibodies, showed nNOS-positive neuronal-like cells (Fig. 2a–f) and NeuN-positive neurons (Fig. 2g, h) in the IG, which could be easily distinguished from the CC in light microscopy by Luxol fast blue and eosin staining (Fig. 1 in Lorenzi et al. 2019). Neuronal NOS-positive nerve fibers were also frequently observed (Fig. 2a–c). In immunofluorescence processed sections, the IG was discriminated from CC by Rhodamine phalloidin staining, evidencing in red both IG and CC nerve fibers with orthogonal orientation (not shown). Double-labeling experiments allowed to show by confocal microscopy that IG nNOS-positive neuronal-like cells were neurons, since they showed immunopositivity both for NeuN (Fig. 3a) and nNOS (Fig. 3b), as confirmed by merging the images (Fig. 3c).

### Morphology and distribution of nNOS–positive neurons in human IG

**Fig. 4:**
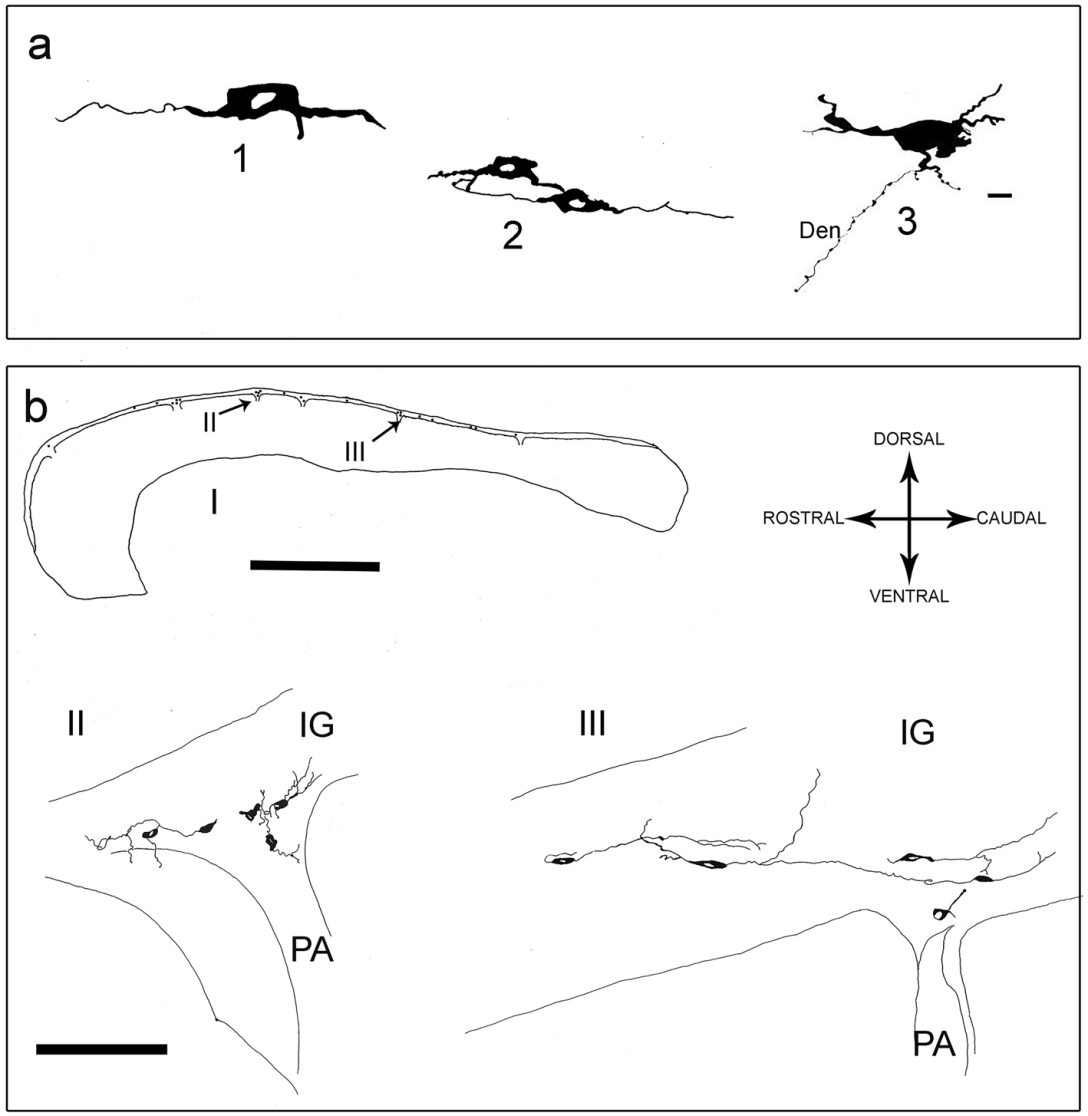
Camera lucida drawings of nNOS-immunopositive neurons in the human IG. **a** Three nNOS positive neurons (ovoidal, 1 and 3; fusiform, 2), one of them showing a long dendrite (Den). **b** Outline figure of CC and overlying IG. The distribution of nNOS-positive neurons in IG is shown (I); two regions (II, III; arrows) are enlarged below in II and III, showing the distribution of nNOS-positive neurons close to pial arterioles (PA). Calibration bars: **a** 10 µm; **b** I, 1 cm; **b** II, III, 100 µm

**Fig. 5:**
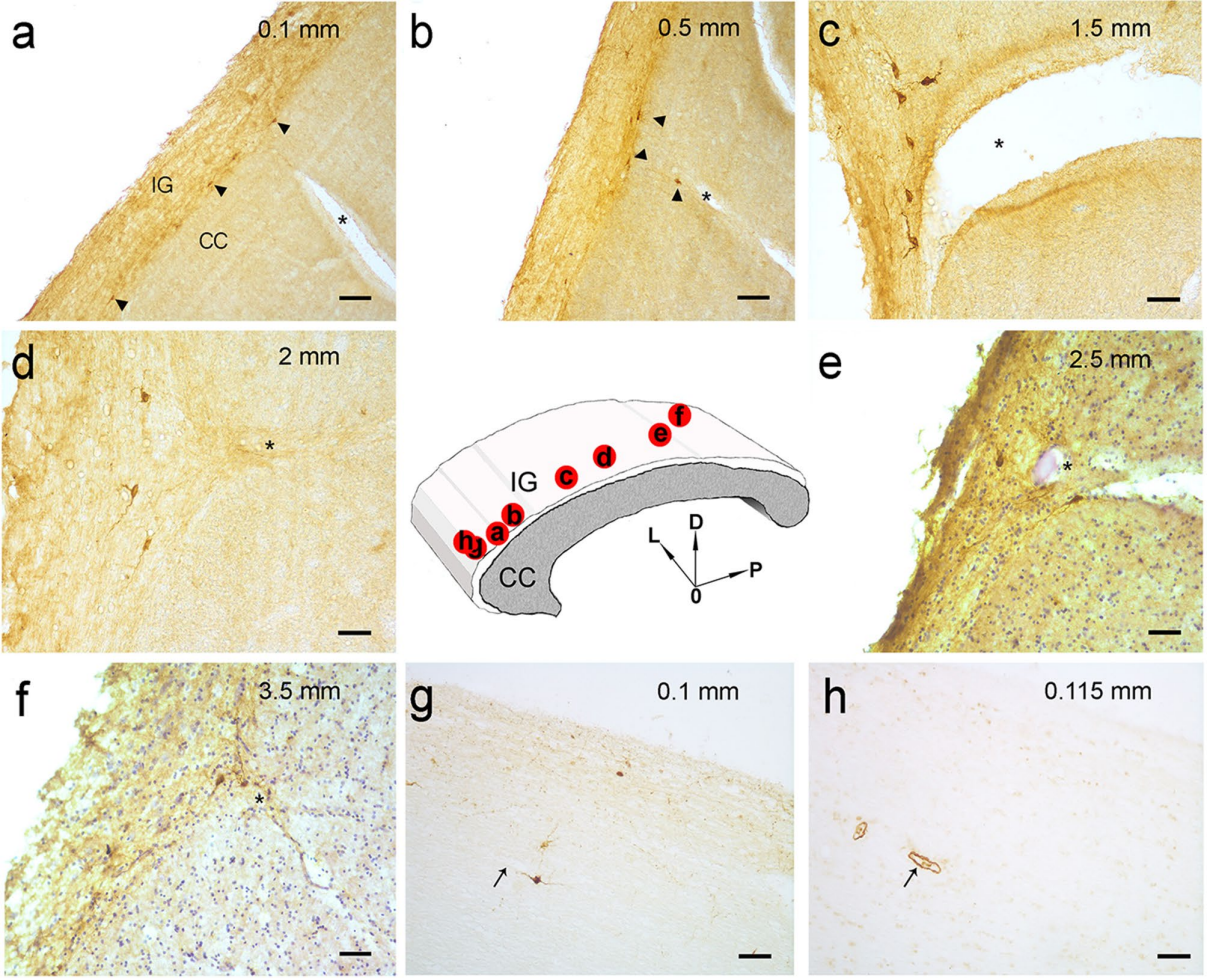
Photomicrographs showing nNOS-positive neurons close to pial arterioles. A schematic representation of CC and overlying IG is shown in the centre. The image in each panel was obtained from sagittal section whose location is indicated by a red circle in the central schema. In the upper right corner of each panel the distance of the section from the midline is indicated. **a**, **b** Low power magnification of nNOS-immunopositive neurons (arrow heads) nearby pial arterioles (asterisks). **c**–**f** Numerous bipolar neurons around pial arterioles (asterisks) at higher magnification. The sections shown in **e** and **f** have been counterstained by hematoxylin to highlight the arterioles. Neurons in proximity of pial arterioles seem to be restricted in the lower portion of IG. **g**, **h** A nNOS-immunopositive neuron and an αSMA-immunopositive arteriole respectively shown in two serial sections: a close association of neuron with the vessel can be observed. *L* lateral, *D* dorsal, *P* posterior direction, *0* midline. Calibration bars: **a**, **b** 100 µm; **c**–**h** 50 µm

**Fig. 6:**
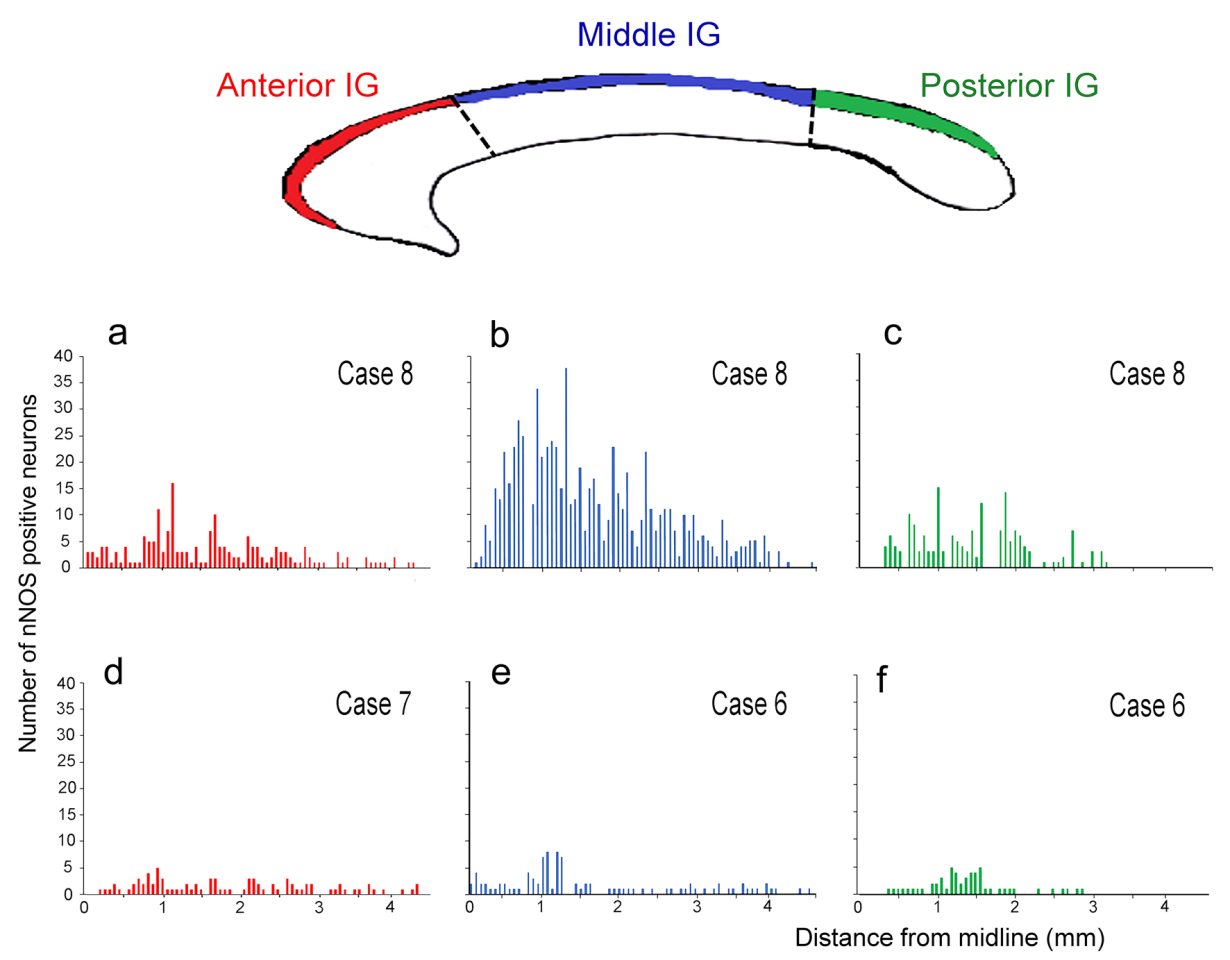
Histograms reporting the distribution of nNOS-immunopositive neurons along the whole human IG (right hemisphere) from three cases (6, 7, 8), as counted in the anterior IG above callosal genu (**a**, **d**), in the middle IG above callosal body (**b**, **e**), and in the posterior IG above callosal splenium (**c**, **f**), along their medio-lateral dimension

**Fig. 7:**
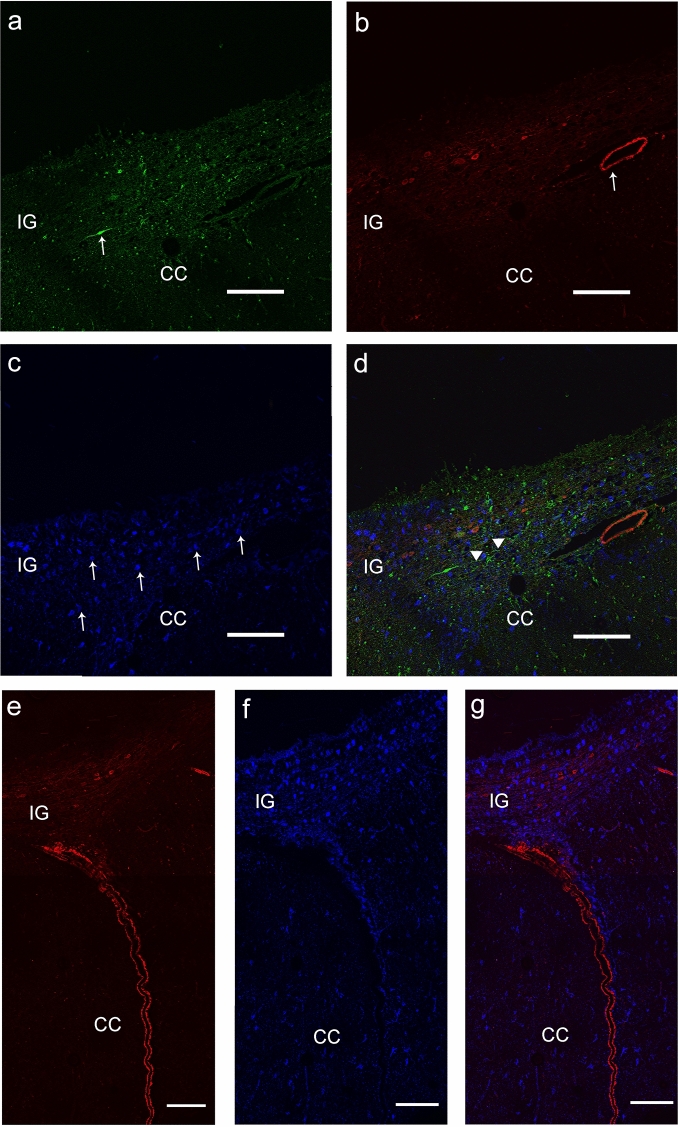
Confocal laser scanning photomicrographs. **a**–**d** and **e**–**g** represent two sagittal sections of human IG. **a** nNOS-positive neuron (green; arrow). **b** αSMA-positive arteriole (red; arrow). **c** GFAP-positive astrocytes (blue; arrows). **d** Merged image. A fiber coming from the nNOS-positive neuron toward the αSMA-positive arteriole is marked by arrow heads. **e** αSMA-positive arteriole (red). **f** GFAP-positive astrocytes (blue). **g** Merged image. Calibration bars: 100 µm

Neuronal NOS-positive neurons were all bipolar, showing mainly fusiform and ovoidal morphologies (Figs. 2 and 4a). They were present throughout the whole thickness of IG, sometimes at the boundary with CC (Fig. 2e, f). Neuronal NOS-positive neurons in sagittal sections were densely packed in proximity to the pial arteries penetrating into the CC, giving rise to a narrow bundle of cells and dendrites (Figs. 4b, II, III, 5a–f). The labeled neurons were generally clustered (Figs. 4b, II, III, 5c, d), although scattered neurons were also observed (Fig. 5a, b). Neuronal NOS positive neurons were located both along rostral-caudal (Table 5; Fig. 4b, I) and medio-lateral (Fig. 6) directions. In general, they were more numerous about 1 mm apart from the midline and over the callosal body (middle IG); in this region, these neurons amounted to 67% of the total number in the whole IG (Table 5, case 8) and their mean number per section was significantly higher than in the anterior and posterior IG (*p* < 0.001; Fig. 6, case 8). Sections reacted with anti-nNOS antibody and stained with ematoxylin-eosin (Fig. 5e, f), as well serial sections reacted with anti-nNOS and anti-α-SMA antibodies (Fig. 5g, h), clearly evidenced the close association of nNOS-positive neurons with vessels. The same close apposition of nNOS-positive neurons with the wall of α-SMA positive vessels was evident in immunofluorescence reacted sections (Fig. 7d). A great number of astrocytes positive to GFAP was also reported in the IG (Fig. 7c, d, f, g), very close to CC penetrating arterioles (Fig. 7g).

## Discussion

The IG is a very small portion of the brain; Tubbs et al. (2013) described the human IG as a glial membrane above the CC without neuronal cells, nor connections with the hippocampus. Recent developmental studies indicate that also human IG contains neurons (Bobić Rasonja et al. 2021), in agreement with a previous Golgi study in rat (Wyss and Sripanidkulchai 1983). Recent studies demonstrated the presence of nNOS-positive neuronal-like cells in human IG, suggesting that it is not a merely rudimentary tissue (Lorenzi et al. 2019; Sanders et al. 2021). The present data confirm earlier findings in adult human IG tissue; in addition, they report, for the first time, a significant presence of nNOS-immunopositive neurons in the IG, suggesting a possible physiological role. In this study, the presence of several nNOS neurons in close proximity to the pial arteries penetrating into the CC was shown, as well as a great number of astrocytes, often very close to the same vessels. The hypothesis proposed is that nNOS neurons and astrocytes in close proximity of arterioles could constitute the IG neurovascular units, participating in the regulation of the CC blood flow. In general, the cerebral blood flow modifies according to the functional activity of the different brain regions (functional hyperemia), so that it increases when the neural activity increases, to guarantee substrate and oxygen delivery, and to remove metabolism by-products, thus maintaining the homeostasis of the cerebral microenvironment (Lassen et al. 1978; Raichle and Mintun 2006; Iadecola 2017). The activity-induced hemodynamic response occurs when neurons, together with astrocytes and vascular cells, communicate through a complex signaling mechanism. These cells act as an integrated unit, termed the neurovascular unit, able to generate and transduce the molecular signals responsible of the changes in blood flow. Brain activation leads to the production of many vasoactive mediators (K^+^, H^+^, neurotransmitters and neuromodulators) which originate from neurons with processes in close contact with blood vessels (Iadecola 2017). Astrocytes are also involved in neurovascular regulation since they have processes in direct contact with both synapses and contractile cells of the vascular wall (Iadecola and Nedergaard 2007). Since a peculiar feature of cerebral circulation is that large cerebral arteries and pial arteries are responsible for two-thirds of the vascular resistance and are therefore the main site of flow control (Faraci and Heistad 1990), IG might have a crucial role in coupling local increases of blood flow in pial branches with metabolic changes related to neuronal function of the underlying CC (Jovanov-Milosevic et al. 2010; Sagrati et al. 2018, 2019). The prevalence of nNOS-positive neurons in the IG overlying the body of CC, which is crossed by sensory-motor fibers where information need to travel fast, further supports the notion that these neurons are involved in modulating the blood flow to face high energy demands. Not by chance, the long callosal artery, one of the main components of the vascular network supplying blood to the CC, gives rise to multiple perforating branches, especially at the level of the body (Kahilogullari et al. 2008). These vessels enter the CC at the midline (Kahilogullari et al. 2008): this observation is consistent with the higher number of nNOS-positive neurons, along the medio-lateral extension, about 1 mm from the midline of the whole IG. The distribution of nNOS neurons in IG seems to be strictly related to the vascular anatomy of CC. The presence of nNOS neurons at the IG/CC boundary points to a role of these neurons in the IG/CC communication, as already hypothesized by recent immunohistochemical findings in rat IG (Barbaresi 2018). These neurons could be similar to those recently observed in layer III of IG which were positive to calbindin (Bobić Rasonja et al. 2021; Sanders et al. 2021), a protein that has a threefold function as buffer, transporter and likely as a non-canonical sensor of Ca^2+^ (Schmidt 2012). It is plausible that these neurons act as neural mediators of signaling between IG and CC, reciprocally enabling to be activated by other brain regions, as indicated by the presence of numerous nNOS-positive fibers in the two structures. Present findings allow to hypothesize that IG is not only an ancillary tissue for the activity of CC, but it is also a real morpho-functional element of the nervous system. This hypothesis is supported by the relevant number of astrocytes observed in the whole IG. Historically considered as merely supporting neurons, recent research has shown that astrocytes actively participate in a large spectrum of central nervous system (CNS) functions including formation, maturation and elimination of synapses, neuronal transmission and modulation of synaptic plasticity (Dallérac and Rouach 2016). The abundance of such multifunction glial cells in IG suggests it is a very active tissue, corroborating the importance of IG in human adult brain.

Other hypotheses have been advanced in recent years suggesting an active functional role for IG. Some studies propose that IG, as a structure containing a heterogeneous mix of both neuronal and glial cells, could have a role in the guidance of the callosal axons during their development (Shu and Richards 2001; Morcom et al. 2016). A very recent study (Izzo et al. 2021) supports the hypothesis of a close interplay between the CC and the IG development. The study describes the hyperplasia of the IG, a midline glial structure, in two rare early gestation fetal cases who also display an abnormally thick and short CC, without any other systemic and/or central nervous system malformation. In addition, in the IG of fetuses with callosal anomalies, NeuN-positive cells were revealed, indicating the presence of neurons, not found in control fetuses; by GFAP immunoreactivity, an increase of glia cells number was also observed respect to the controls, further suggesting that an abnormal IG aspect could be associated to an altered CC embryological development.

Another recent study proves that IG has its own distinct histogenetic differentation pattern (Bobić Rasonja et al. 2021), and it does not shows signs of regression during the fetal period; these observations suggest that IG is not a rudimentary tissue, but it plays a functional role in the adult brain. In line with this hypothesis, NeuN-positive cells, i.e. neurons, have been found in the IG (Bobić Rasonja et al. 2021), both during fetal development and in adults, in accordance with present research.

## Conclusion

In conclusion, these novel findings (summarized in Graphical Abstract) shed new light on IG which, rather than an undifferentiated part of the hippocampal formation, can be considered an anatomo-functional structure with a key role in the neurovascular regulation within the CC. Considering that the CC represents a hallmark of brain development, our results provide indications for future studies aimed at better understanding the physiology of IG and at investigating any potential involvement of this structure in neurological disorders.

## Data Availability

The datasets, material and code that support the findings of the current study are available from the corresponding author upon reasonable request. The data are not publicly available due to privacy or ethical restrictions.
